# IgG SARS-CoV-2 Antibodies Persist at Least for 10 Months in Patients on Hemodialysis

**DOI:** 10.1016/j.ekir.2021.03.900

**Published:** 2021-04-08

**Authors:** Caroline Dudreuilh, Tayeba Roper, Cormac Breen, Paramit Chowdhury, Sam Douthwaite, Nicola Kumar, Dimitrios-Anestis Moutzouris

**Affiliations:** 1 Renal & Transplant Unit, Guy's and St. Thomas' NHS Foundation Trust, London, UK; 2 Department of Infectious Diseases, Guy's and St. Thomas' NHS Foundation Trust, London, UK

See Commentary on Page 1761

Patients with kidney failure have an impaired response to vaccination,^1^ and an increased susceptibility to infections.^2^ We have previously shown that the preventive and isolation measures in our dialysis units have had a positive impact, slowing the spread of coronavirus disease 2019 (COVID-19), and protecting our hemodialysis (HD) patients while maintaining optimal dialysis treatment.^3^^,^^4^ However, patients with kidney failure who were diagnosed with COVID-19 were at significantly higher risk of hospital admission and death.^5^

Previous reports have shown up to 30% of patients on hemodialysis to have immunoglobulin G (IgG) antibodies positive for severe acute respiratory syndrome coronavirus 2 (SARS-CoV-2), some without history of symptoms or positive screening tests.^6^, ^7^, ^8^ In healthy individuals, recent reports suggest that the presence of SARS-CoV-2 IgG antibodies lasts up to 8 months.^9^^,^ S1^,^S2 It has been suggested that the severity of COVID-19 is correlated with the capacity to produce a strong and sustained IgG respons.S3 This has not been confirmed in patients on HD.

The aim of our study was to examine the persistence of SARS-CoV-2 IgG antibodies in patients on HD over time.

## Methods

Guy’s and St. Thomas’ NHS Foundation Trust provide HD in two hospital-based dialysis units and six satellite units for 697 patients currently. Patients who consented in July 2020 (T0) were tested for the presence of IgG specific against SARS-CoV-2 (LIAISON SARS-CoV-2 S1/S2 IgG, targeting the spike, DiaSorin, Saluggia, Italy). Clinical data and biological data were collected prospectively up to January 2021. During follow-up, all the patients on HD were screened weekly with reverse-transcriptase polymerase chain reaction (RT-PCR) nasopharyngeal swab specific for SARS-CoV-2 (Aptima, SARS-CoV-2 Assay [Panther System], Hologic, San Diego, CA), as part of our preventive strategy.^3^^,^^4^ In January 2021 (T1), all patients enrolled in July 2020 were tested for the presence of IgG against SARS-CoV-2. This study was approved by the local ethics committee. All studies were conducted in accordance with the Declaration of Helsinki. See supplementary Methods for statistical analysis.

## Results

**Figure 1:**
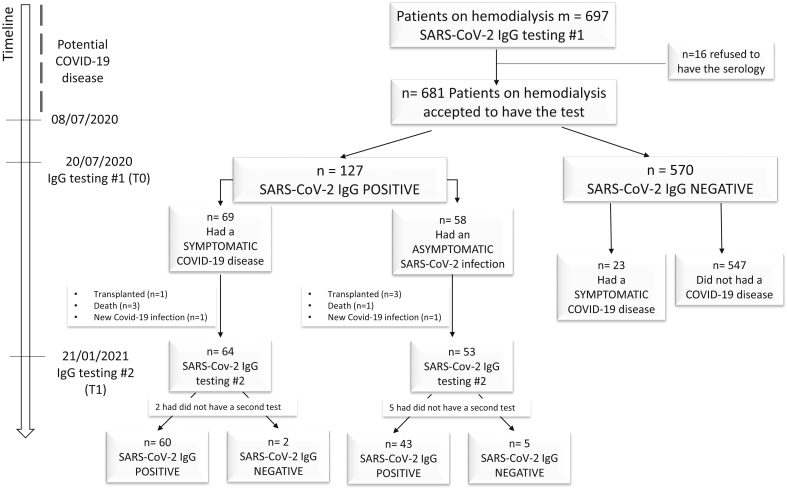
Flow-chart of patients on hemodialysis tested for presence of immunoglobulin G (IgG) specific for severe acute respiratory syndrome coronavirus 2 (SARS-CoV-2) in July 2020. COVID-19, coronavirus disease 2019.

Among the 697 patients on HD in July 2020, 127 patients (18%) were positive for IgG SARS-CoV-2 antibodies. Sixty-nine of the 127 IgG-positive patients (54%) have had a symptomatic COVID-19 illness, whereas 58 (46%) had an asymptomatic illness. Twenty-eight of 69 symptomatic patients (41%) (see Supplemental Table S1) were admitted to the hospital including 4 (12.5%) who required an intensive care admission. Five hundred seventy patients did not have detectable IgG antibodies against SARS-CoV-2, including 23 patients who had a confirmed COVID-19 infection (Figure 1) by RT-PCR. For those who had a symptomatic COVID-19 infection, the average time between the COVID-19 disease and T0 was 112 (±48) days.

In January 2021, from the group of 127 IgG antibody–positive patients, 4 patients died from non–COVID-19–related causes, 4 received a kidney transplant, and 2 patients had a new asymptomatic SARS-CoV-2 infection confirmed thanks to the weekly RT-PCR screening in all our units (Figure 1). These two patients were diagnosed on a systematic RT-PCR screening 161 days after T0 and were totally asymptomatic at the time of diagnosis. One of them had a previous confirmed COVID-19 infection 304 days before this infection, and the other one had an asymptomatic first SARS-CoV-2 infection.

In January 2021, of the 117 patients on HD, 110 were tested for IgG antibodies (94%). One hundred three of 110 patients (94%) had persistent detectable IgG against SARS-CoV-2. Sixty of 62 patients with a previous symptomatic COVID-19 disease had detectable antibodies (96.8%). Forty-three of 48 patients who had a previous asymptomatic COVID-19 had detectable antibodies (89.6%). For patients who had a symptomatic COVID-19, the mean time between the infection and the second SARS-CoV-2 IgG antibody test was 9.8 ± 0.53 months.

In January 2021, of 110 patients who had detectable IgG against SARS-CoV-2 at T0, 7 patients had no detectable IgG against SARS-CoV-2 (6%) at T1. The clinical characteristics of these patients are presented in Table 1. Patients on immunosuppression included patients on low-dose steroids or tacrolimus. There were no patients with recent (less than 1 year) exposure to high-dose steroids or biological agents. Twenty-nine percent of patients on immunosuppression had undetectable antibodies compared with 7% of patients who were not on immunosuppression; however, this did not reach statistical significance given the limited numbers.

**Table 1:** Clinical Characteristics of Patients on Hemodialysis With a Positive SARS-CoV-2 Test in July 2020 (T0), Based on Serology Results in January 2021 (T1)^a^^,^^b^

Characteristics	SARS-CoV-2 IgG–Positive at T1 (n = 103)	SARS-CoV-2 IgG–Negative at T1 (n = 7)	*P*
Patient at T0			
Age, years (median)	63 (23-90)	64 (42-81)	0.73
Male; female	57 (55); 46 (45)	4 (57); 3 (43)	1
Ethnicity			
Black	59 (57)	3 (43)	0.7
Caucasian	25 (24)	3 (43)	0.36
Asian	8 (8)	1 (14)	0.46
Other	11 (11)	0	1
Cause of KF			
Hypertension	32 (31)	1 (14)	0.67
Diabetes	44 (43)	3 (43)	1
Glomerulopathy	11 (11)	0	1
COVID-19–related	2 (2)	0	1
Others/unknown	10 (9) / 4(4)	2 (29)/1 (14)	0.24 /0.28
Time on hemodialysis, months (median)	31 (0-384)	31 (2-204)	0.67
Previous transplantation	16 (16)	2 (29)	0.32
Currently immunosuppressed	7 (7)	2 (29)	0.1
HIV	6 (6)	0 (0)	1
First COVID-19 illness			
Time between T1 and first positive RT-PCR for SARS-CoV-2, months, mean (SD)	9.8 ± 0.53	10 ± 0.6	0.49
Symptomatic; asymptomatic	60 (58); 43 (42)	2 (29); 5(71)	0.23
Admission	26 of 60 (43)	1 of 2 (50)	1
ICU admission	4 of 26 (15)	0 (0)	1

a COVID-19, coronavirus disease 2019; HIV, human immunodeficiency virus; ICU, intensive care unit; IgG, immunoglobulin G; KF, kidney failure; Others, includes autosomal polycystic kidney disease, urological causes and other causes not presented; RT-PCR, reverse-transcriptase polymerase chain reaction; SARS-CoV-2, severe acute respiratory syndrome coronavirus 2.

b Values shown are n (%) unless otherwise noted.

## Discussion

In a cohort of 127 patients on HD who had detectable IgG against SARS-CoV-2 in July 2020, 94% of the patients had persistent detectable IgG titers 6 months later. Only two patients were found to be re-infected during the follow-up. SARS-CoV-2 IgG antibodies are detected at least up to approximately 10 months in patients on HD with a previous symptomatic infection. To our knowledge this is the longest follow-up of SARS-CoV-2 serology in a population on HD.

The degree of protection against COVID-19 provided by the presence of IgG against SARS-CoV-2 remains unclear. In recent reports in nonimmunocompromised individuals,^9^^,^S1^,^S2 the presence of IgG against SARS-CoV-2 seems to give protection against re-infection up to 8 months. However, preliminary data in a kidney transplant populationS4 showed that nearly 64% of the patients lost the IgG response to SARS-CoV-2 6 months after the symptomatic infection. In the current study, we showed that the majority of patients with a positive serology in July had a sustained IgG response at 6 months. Seven patients had no detectable IgG antibodies after 6 months, but they were not found to be re-infected during the follow-up.

Our study has several limitations mainly reflecting the policy changes during the COVID-19 pandemic. For patients who had an asymptomatic infection it was not possible to assess the time from the infection episode as we have implemented the weekly RT-PCR testing in our patients on HD patients since April 2020. Before this, we were testing only symptomatic patients. We assume that asymptomatic patients were infected before April 2020. Therefore, even in this group of asymptomatic patients, 89.6% had persistent IgG antibodies at least 10 months after the infection (and possibly longer). In addition, our analysis would have benefited from a more in-depth analysis of the humoral response to COVID-19, including anti-receptor binding domain/anti-nucleoprotein antibodies. The LIAISON SARS-CoV-2 S1/S2 IgG test presents a sensitivity of 97.4%.S5 It would have been interesting to evaluate the cellular response to SARS-CoV-2 patients, particularly in patients who did not have detectable antibodies at the last follow-up, but they were not found to be re-infected. The results of the genetic testing of the SARS-CoV-2 were not available for the patients who were found to be re-infected during follow-up; therefore, we cannot rule out that the re-infection was associated with the new B.1.1.7 variant. Finally, the numbers of the patients on HD in the different subgroups (*e.g.*, patients on immunosuppression or with a previous kidney transplant) were small.

Our study showed that the majority of our patients on HD had persistent IgG SARS-CoV-2 antibodies at least for 6 months after they were first detected. Weekly systematic RT-PCR screening helped to identify the only two patients who presented with re-infection during the follow-up and who were both asymptomatic. Longer follow-up time in larger dialysis populations and more detailed evaluation of the immune response would be helpful in designing future vaccination policies and implementation of isolation and de-isolation measures in dialysis units.

## Supplementary Material

Supplementary File (PDF)

**Table S1.** Clinical characteristics of patients on hemodialysis with a positive IgG SARS-CoV-2 test in July 2020 (T0).

**Supplementary Methods**

**Supplementary References**
