# Supplementary material for: IgG SARS-CoV-2 Antibodies Persist at Least for 10 Months in Patients on Hemodialysis
Source: Kidney Int Rep. 2021 Apr 8;6(7):1961–4. doi: 10.1016/j.ekir.2021.03.900 (PMC8028697; doi:10.1016/j.ekir.2021.03.900)
Supplement: Supplementary File (PDF) [file mmc1.pdf]

Supplementary material

Supplementary methods:

Local SARS-CoV-2 RT-PCR screening policy

As previously published[3], since the beginning of the pandemic, patients on hemodialysis were routinely screened weekly for asymptomatic SARS-CoV-2 infection with RT-PCR. Positive patients were isolated in a dedicated dialysis unit and had a repeat RT-PCR 7 days after the positive swab. They were discharged to their usual dialysis unit following two consecutive negative RT-PCR.

Statistical analysis

Results are expressed as percentage or mean ± SD for normally distributed variables and median (range) for non-normally distributed variables. Categorical data were compared by chi-square or Fisher's exact test according to sample size. Quantitative data were compared by Mann-Whitney test. Statistical analyses involved using XLSTAT software. A $p<0.05$ was considered statistically significant.

Supplemental Table S1. Clinical characteristics of patients on hemodialysis with a positive IgG SARS-Cov-2 test in July 2020 (T0).

**Patients' characteristics**		**n= 73 Symptomatic Covid-19 infection**	**n= 54 Asymptomatic Covid-19 infection**	*p*
Age, years (median)		61 (23-85)	67 (28-90)	***0.048***
Gender, male (%) / female (%)		40 (55), 33 (45)	34 (63), 20 (37)	*0.37*
Ethnicity, n(%)	Black	35 (48)	32 (59)	*0.21*
	Caucasian	21 (29)	14 (26)	*0.84*
	Asian	5 (7)	2 (4)	*0.7*
	Other	12 (16)	6 (11)	*0.45*
Cause of KF, n(%)	Hypertension	23 (32)	15 (28)	*0.69*
	Diabetes	28 (38)	26 (48)	*0.28*
	Glomerulopathy	6 (8)	7 (13)	*0.39*
	Covid-19-related	2 (3)	0 (0)	*0.51*
	Others /Unknown	8 (11) / 6 (8)	5 (9) /1 (2)	*0.77 / 0.23*
Time on haemodialysis, months (median)		28.5 (0-384)	32 (1-221)	*0.88*
Previous transplantation		17 (23)	5 (9)	*0.056*
Current immunosuppression		7 (10)	7 (13)	*0.47*
HIV		5 (7)	2 (4)	*0.7*
**Covid-19 illness characteristics**				
Time between serology and first positive RT-PCR for SARS-Cov-2, days, mean (SD)		112 ± 48	N/A	
Admission		32 (31)	N/A	
	ICU Admission	4/32 (12.5%)	N/A	

IgG, immunoglobulin G ; KF, kidney failure; APKD, autosomic polycystic kidney disease ; RT-PCR, reverse-transcriptase polymerase chain reaction ; N/A, not applicable; ICU, intensive care unit ; HIV, human immunodeficiency virus

Others: includes APKD, Urological causes and other causes not presented in the table

Supplementary References

S1. Rodda LB, Netland J, Shehata L, Pruner KB, Morawski PA, Thouvenel CD, et al. Functional SARS-CoV-2-Specific Immune Memory Persists after Mild COVID-19. Cell. 2021 Jan;184(1):169-183.e17.

S2. Ripperger TJ, Uhrlaub JL, Watanabe M, Wong R, Castaneda Y, Pizzato HA, et al. Orthogonal SARS-CoV-2 Serological Assays Enable Surveillance of Low-Prevalence Communities and Reveal Durable Humoral Immunity. Immunity. 2020 Nov;53(5):925-933.e4.

S3. Ibarrondo FJ, Fulcher JA, Goodman-Meza D, Elliott J, Hofmann C, Hausner MA, et al. Rapid Decay of Anti–SARS-CoV-2 Antibodies in Persons with Mild Covid-19. N Engl J Med. 2020 Sep 10;383(11):1085–7.

S4. Chavarot N, Leruez-Ville M, Scemla A, Burger C, Amrouche L, Rouzaud C, et al. Decline and loss of anti–SARS-CoV-2 antibodies in kidney transplant recipients in the 6 months following SARS-CoV-2 infection. Kidney International. 2021 Feb;99(2):486–8.

S5. Public Health England. Evaluation of DiaSorin LIAISON SARS- CoV-2 S1/S2 IgG serology assay for the detection of anti-SARS-CoV-2 antibodies . https://assets.publishing.service.gov.uk/government/uploads/system/uploads/attachment_data/file/893435/Evaluation_of_Diasorin_Liaison_anti_SARS_CoV_2.pdf
